# The early peak knee abduction moment waveform is a novel risk factor predicting anterior cruciate ligament injury in young athletes: A prospective study

**DOI:** 10.1002/ksa.12471

**Published:** 2024-09-12

**Authors:** Haraldur Björn Sigurðsson, Melkorka Katrín Fl Pétursdóttir, Kristín Briem

**Affiliations:** ^1^ Department of Physical Therapy, Faculty of Medicine University of Iceland Reykjavik Iceland; ^2^ Research Centre of Rehabilitation and Movement Science University of Iceland Reykjavik Iceland

**Keywords:** ACL rupture, biomechanics, cluster analysis, injury prevention, longitudinal data, motion analysis

## Abstract

**Purpose:**

In this study, prospective data were used to evaluate whether the early peak knee abduction moment waveform is associated with the risk of anterior cruciate ligament (ACL) injury.

**Methods:**

Biomechanical data from 84 athletes who participated in the study as adolescents were analysed after cross‐referencing national health registry data to confirm ACL reconstruction in the subsequent years. The knee abduction moment waveform shape was obtained with cluster analysis for the first 100 ms of a cutting manoeuvre (1776 trials in total) and classified as either containing an early peak knee abduction moment or not, and the odds ratio for later ACL injury was then calculated. Additionally, discrete kinematic and kinetic variables were extracted, and tested against the risk of ACL injury using mixed model logistic regression.

**Results:**

Of 84 athletes, 8 (all female) sustained a total of 13 ACL injuries in the years after motion analysis data collection. Six clusters of knee abduction moment waveform shapes were identified. Two clusters containing 446 trials were classified as an early peak knee abduction waveform. This waveform was associated with a 7.2‐fold increase in the risk of ACL injury (95% confidence interval: 2.4–24.6; *p* < 0.001). Of the kinematic and kinetic variables tested, only the knee abduction angle at initial contact was associated with an increased risk of ACL injury (*p* < 0.001).

**Conclusion:**

This is the first study to confirm the association between the early peak knee abduction moment waveform and the risk of ACL injury. Using waveforms, instead of discrete peak values of the knee abduction moment, may better represent risky movement patterns. Replicating these findings in a larger cohort will support the use of this method to screen athletes for risk and guide targeted preventive interventions and their efficacy.

**Level of Evidence:**

Level II.

**Abbreviations**
ACL: anterior cruciate ligament
CI: confidence interval
KAM: knee abduction moment
OR: odds ratio
QTM: Qualisys track manager
vGRF: vertical ground reaction force

## INTRODUCTION

Anterior cruciate ligament (ACL) injuries are both very serious and fairly frequent. The seriousness derives from long‐term reductions in quality of life [9], the high cost of treatments [21] and detriments to knee health [1]. At an overall incidence rate of 75 per 100,000 person‐years [25], the accumulated costs of ACL injuries amount to quite a societal burden, especially since the incidence is the highest among individuals in their late teens and early 20s [25]. For these reasons, researchers continue to chase the dragon of ACL injury prevention.

A key component of preventive research is establishing modifiable risk factors to devise preventive strategies. Numerous such studies have investigated biomechanics as a risk factor for ACL injury. Among the variables studied is the knee abduction moment [14, 18, 19]. One prominent prospective study found the knee abduction moment identified during the stance phase of a bilateral drop‐jump task to be predictive of injury [14], but two later studies have not replicated the results [18, 19], resulting in ambiguity.

There are good reasons to believe that the knee abduction moment is a risk factor for injury, and that the aforementioned studies are not a valid test of the risk factor hypothesis. These reasons emerge from the injury mechanism literature. Cadaveric model studies leave little doubt that the knee abduction moment is part of the ACL injury mechanism [4, 5]. Video analyses of actual injury situations also indicate that there is likely a knee abduction moment component to the injury, although without force plates, these studies obviously lack kinetic data [7, 16, 17]. What has also been revealed is the timing of the injury, which is estimated to happen around 50 ms after ground contact [17].

It is from the injury timing that a key discrepancy between most risk factor studies and injury mechanism studies emerges. Biomechanical risk factor studies have examined peak values extracted from either the entire landing phase or the whole stance phase [14, 18, 19]. However, this length of time far exceeds the timing of ACL injuries; additionally, the vertical drop‐jump task is bilateral, whereas the injury mechanism is primarily single‐leg [8, 16, 20].

To address these shortcomings, methods have been developed by the research group of the authors of the current manuscript, to enable analyses that are consistent with the mechanism and timing of ACL injury and within the limitations of the technology being used. The result of this labour is a cluster analysis method that classifies movement trials according to the waveform shape, specifically identifying whether they contain an early peak knee abduction moment or not [31]. Later work using this method has shown that the early peak knee abduction moment is related to kinematics that is in turn associated with injury [33], and that the early peak knee abduction moment is one potential mechanism through which intervention programmes work [32]. However, whether the occurrence of early peak knee abduction moment during sporting movements is related to the risk of suffering an actual ACL injury or not has not been verified.

The primary aim of this study was to evaluate whether the frequency of early peak knee valgus moment waveforms, identified during repeated trials of a change of direction task in a cohort of adolescent athletes, is associated with the risk of later sustaining an ACL injury. The secondary aim was to analyse whether movement patterns reflecting dynamic valgus or stiff landings are associated with the risk of ACL injury.

## METHODS

This cohort was previously recruited for a prospective study, and the data were used for several publications [6, 10, 26, 31, 33]. Four years after the conclusion of the parent study, ethical approval was granted by the National Ethics Committee (VSN, approval code: 12‐040‐V8) to contact the participants to have them reconsent to the analyses of this study. Information from the national health registry was used to obtain confirmation of any ACL reconstruction involving consenting participants.

Because of the nature of the study, very few controls are in place. Information about continued sports participation after lab testing or the training load experienced by the athletes is not available. Because the purpose of the original study was not to examine the risk of actual ACL injuries, this study should be considered as hypothesis generating rather than hypothesis testing.

### Subjects

In the original study, 277 youth athletes (aged 9–12) participating in soccer or team handball were enroled between 2012 and 2014; 5 years later (during 2017–2019), 177 of these athletes, now in their teens, agreed to a follow‐up lab visit. Motion analysis data from this second lab visit were used for the present study. In the fall of 2023, 138 of these 177 participants with follow‐up data consented to have their medical records reviewed for a history of knee surgery and to have their motion analysis data re‐analysed. Of these consenting participants, 84 (53 females) were actively participating in their chosen sport at the time of the second data collection, had valid biomechanical data and were included in the analysis. Data from non‐athletes were not used for the analysis due to the focus on risk factors associated with sports participation.

National health registry data confirmed that nine athletes (all female) had knee surgeries during subsequent years, of whom eight had undergone ACL reconstruction surgeries. All of them filled out an online form using the REDCap system [12, 13], providing information regarding which leg was injured during each instance (if more than one) and the circumstances of each ACL injury (contact or non‐contact).

### Baseline measurements

**Figure 1:**
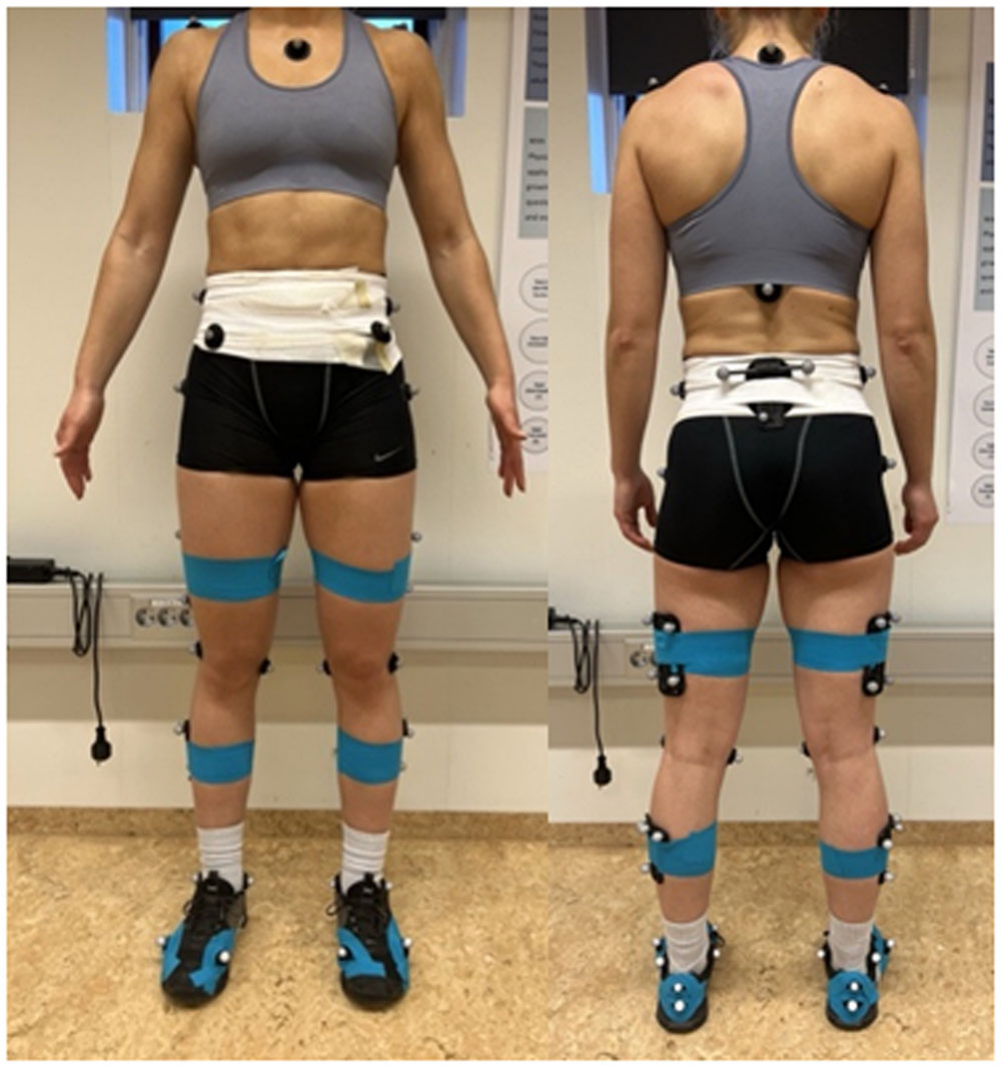
Marker set‐up.

At baseline, athletes' height and weight were measured before a 5‐min warm‐up on a stationary bicycle at a self‐selected pace. Athletes wore athletic shorts and a sports bra and used either their own shoes or borrowed sneakers from the lab. Forty‐six retro‐reflective markers were placed on the athlete to define and track the feet, shanks, thighs, pelvis and trunk, according to the lab's protocol (Figure 1) [6, 10, 26, 31, 33, 34]. An eight‐camera system (Qualisys AB) was used to capture motion data at a resolution of 0.3 MP and a sampling frequency of 400 Hz. Ground reaction force data were collected using AMTI force plates, sampling at 2000 Hz. Joint centres of rotation were determined as the mid‐point between segment markers (except for 25% of the distance between greater trochanters for the hips) using a static trial after which anatomical markers on the malleoli, femoral epicondyles, greater trochanters and iliac crests were removed.

**Figure 2:**
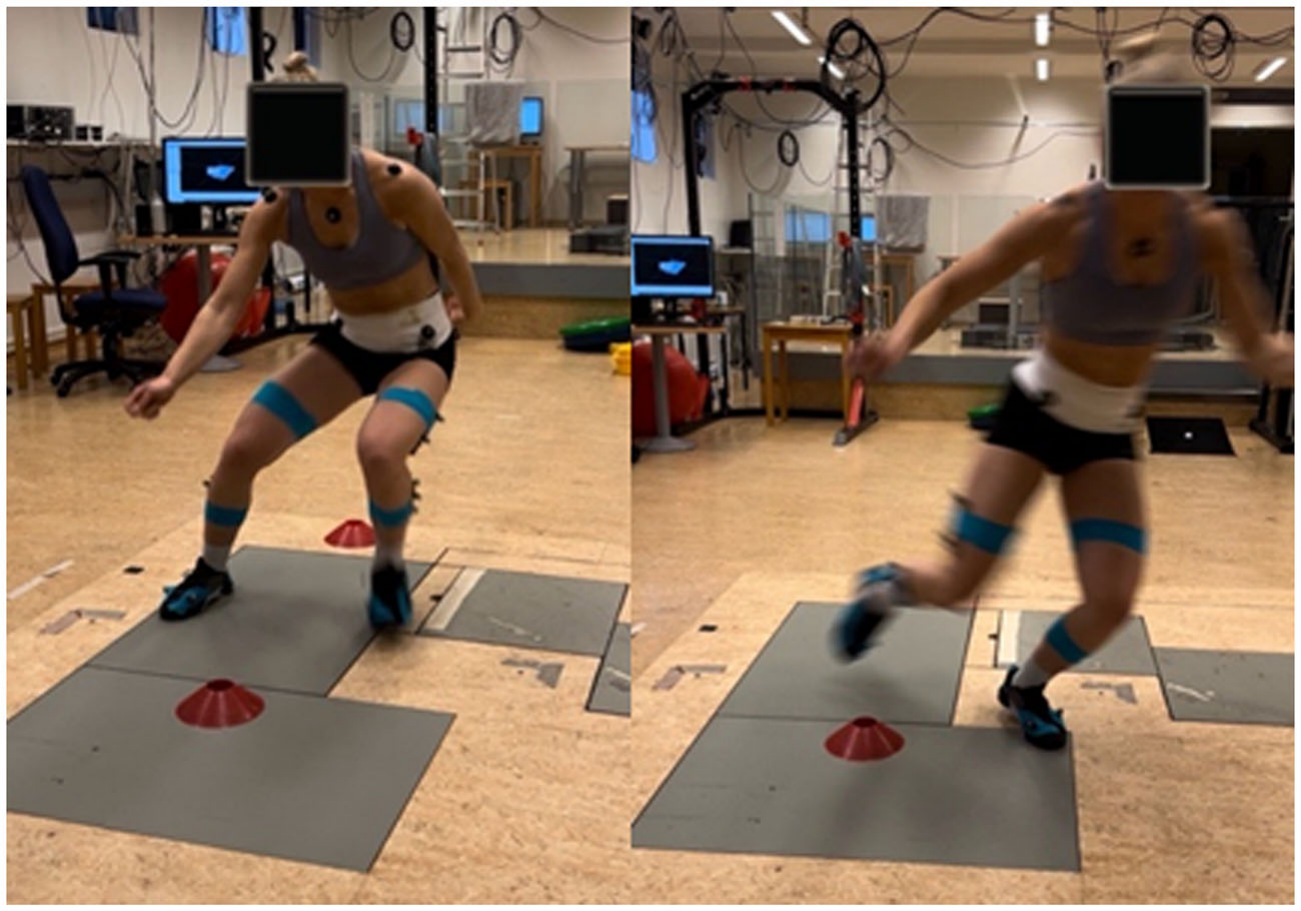
Cutting manoeuvre.

The athletes performed five repetitions of a bilateral drop jump (not further analysed here) and a cutting manoeuvre (five to each side) in a randomized order. The cutting manoeuvre used self‐selected techniques with the aim of getting past a stationary object representing an opponent. The angle of the manoeuvre was not further standardized, and the athletes started from a ready position with no run‐up. All manoeuvres were repeated after a 5‐min lateral slide‐board exercise, resulting in a total of 10 repetitions per leg (Figure 2).

### Data processing

Markers were labelled using the QTM software (Qualisys AB) and exported as c3D files for further processing in Visual3D (C‐motion). Kinematics were calculated using 6DOF segments. Kinetics were calculated using inverse kinematics, where each joint was resolved in the more proximal segment. The resulting kinetics and kinematics were low‐pass filtered at a 6 Hz cut‐off frequency. The frequency chosen was based on the clustering algorithm, which works best with simplified curve shapes [31]. The start (initial contact) and end of stance were defined as the first frame where the vertical ground reaction force exceeded 10 N and fell below 10 N, respectively. The knee abduction moment was defined such that the positive values indicated an external abduction moment, and the negative values indicated an external adduction moment. The moment was normalized to body mass (N m/kg). All joint angles are reported in degrees.

### Variable definitions

#### Early peak waveform

The knee abduction moment during the first 100 ms after initial contact was classified as either an early peak or not, using a clustering algorithm as previously described [31]. In short, the waveforms were reduced to the sign of the differentiated curve and the Euclidean distance between each trial was calculated. The clusters were formed using the Ward.d2 algorithm [24]. The goodness of fit of the cluster solution was calculated using the c‐index, which represents the ratio of within‐cluster distances to between‐cluster distances.

#### Early stance knee abduction loading

The variables used to represent the early stance knee abduction loading consisted of the highest knee abduction moment (N m/kg) within 100 ms of initial contact, the knee abduction angle at initial contact and the distance between the trunk and foot centre of mass in the anteroposterior and mediolateral directions (as a percentage of thigh length) at initial contact. Taken together, these variables were used to represent, in part, what has been termed the dynamic valgus movement pattern [27]. Other variables commonly associated with the dynamic valgus movement pattern such as the hip adduction and internal rotation ultimately affect the ACL through kinetics and kinematics of the knee joint. The knee abduction moment is the primary variable that connects the movement pattern to potential injury risk as part of the injury mechanism [5], whereas the knee abduction angle and trunk–foot distance would primarily influence the knee abduction moment by increasing the length of the moment arm.

Although the early stance phase is the timeframe during which ACL injuries occur [17], an interesting recent prospective risk study used the peak knee abduction moment over the entire stance phase for their analysis [22]. In the interest of comparing our results to that paper, this variable was also analysed albeit with different signal filters.

#### Stiff landing movement pattern

The stiff landing movement pattern was defined as the knee flexion angle at initial contact and the highest vertical ground reaction force within 100 ms [19].

### Statistical analysis

As only eight participants in the study had ACL injuries, the risk of false positive results increases quickly as the number of included variables increases [29]. Therefore, statistical analyses were performed separately for each discrete kinematic and kinetic variable. A Bonferroni adjustment was applied to the *p* values for each family of related variables. Discrete knee moments and the vGRF formed one group of variables, whereas the sagittal and frontal knee kinematics with trunk–foot distance formed the other. A mixed model logistic regression [2] was performed with repeated measures as the random effect and each variable of interest as the only fixed effect. The alpha was set at 0.05, and *p* values for fixed effects were calculated with an ANOVA, comparing each model to a base model without the fixed effects using a chi‐squared test.

The early peak waveform is the primary variable of interest in this study. Therefore, we went to great lengths to explore the validity and robustness of the results regarding the early peak waveform. To explore the stability of the model parameters and whether the results were overly affected by outliers, a leave‐one‐out cross‐validation was performed. During this step, a logistic regression was calculated for each of the injured athletes where that athlete was excluded, resulting in eight logistic regressions. The *p* values and the odds ratios (PRs) were extracted from each and reported separately. The observed power was also calculated, as it could not be calculated a priori. For this, we used the SIMR package [11] to perform repeated simulations and estimate the power of the hypothesis test.

## RESULTS

The eight athletes sustained a total of 13 ACL injuries: eight on the right side and five on the left side. Five athletes sustained a single injury, two sustained a total of three injuries each and one sustained two injuries. All ACL injuries were non‐contact. The mean (range) time from data collection to (first) surgery was 32 (8–70) months (data available for *n* = 7). The mean (range) time from data collection to the end of the study period for non‐injured athletes was 59 (48–78) months.

### Early peak waveform

**Figure 3:**
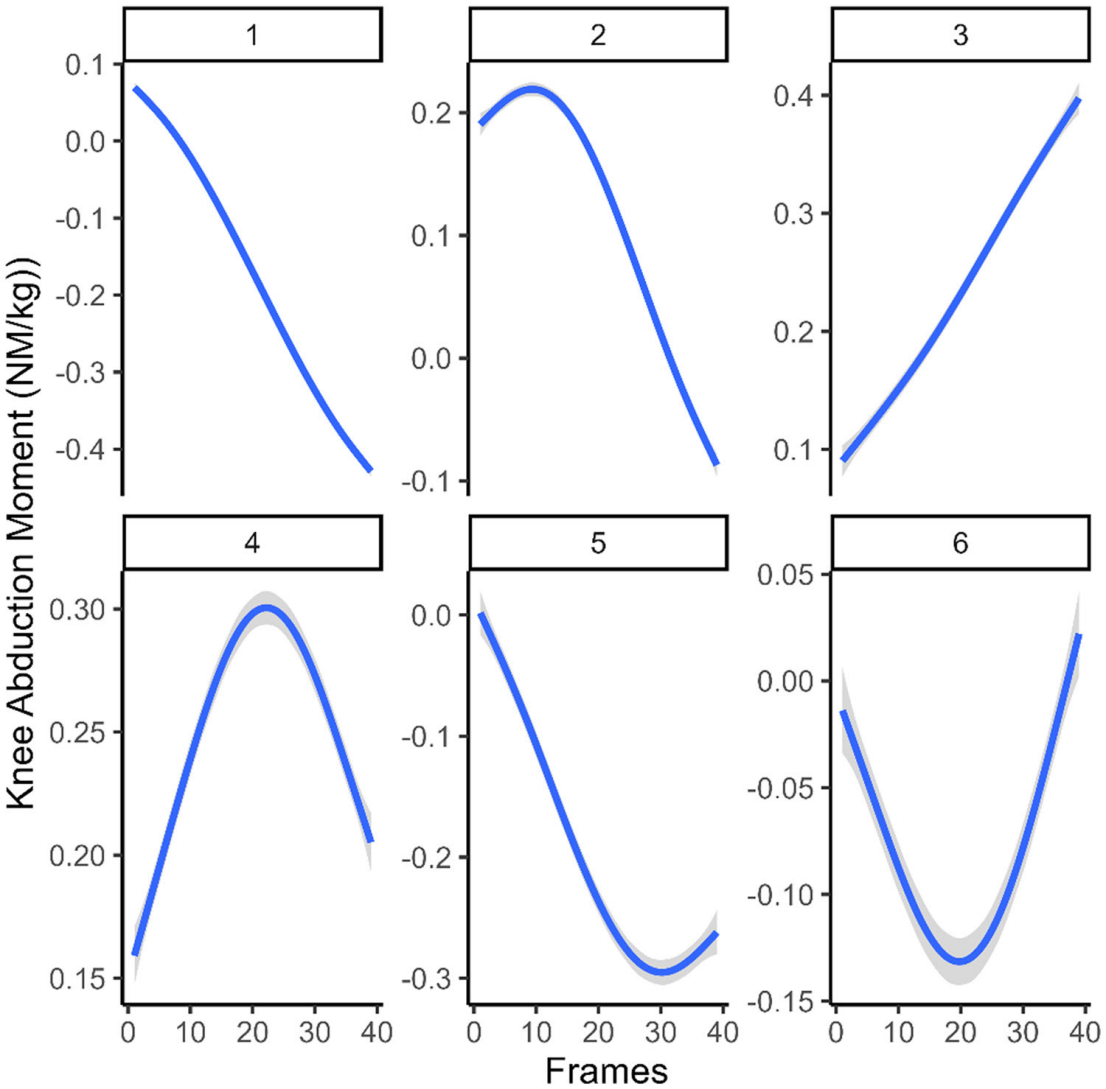
Early knee abduction moment waveforms. Waveforms labelled 2 and 4 were classified as early peaks, whereas Waveforms 1, 3, 5 and 6 were classified as others. The shaded area shows the 95% confidence interval of the smoothing function. Note that the first 39 frames (less than 100 ms) were used for cluster analysis.

A total of 1776 trials from the 84 included participants were entered into a clustering algorithm, resulting in the formation of six clusters with a c‐index of 0.15 (Figure 3). Two of the six clusters (446 trials) were classified as early peak waveforms with a visual inspection of the average curve shape, whereas the other shapes were not further differentiated (1330 trials).

An early peak knee abduction moment waveform was associated with a 7.2‐fold increase in the risk of ACL injury (95% confidence interval [CI]: 2.4–24.6, *p* = 0.00025, *r* ^2^ = 0.021). Observed power was 93% (95% CI: 91.3%–94.6%). During cross‐validation, the effect was always present, with *p* values ranging from 0.03 to 0.0001 and ORs ranging from 3.8 to 20.5 (Table S1).

### Other variables

The results from the other variables are listed in Table 1. The mean (SD) timing of the early KAM was 22 (31) ms after initial contact (8.6 [12.5] frames), whereas the highest KAM timing was at 317 (82) ms (126.9 [32.9] frames). In only 51 trials (0.03%), the early and highest KAM were the same event. Only the knee abduction angle at initial contact was associated with the risk of ACL injury.

**Table 1:** Results of mixed logistic regressions for discrete values of specific variables.

Variable	95% CI				
	OR	Lower	Upper	*p* Value	*r* ^2^
Knee abduction angle (°)	1.4	1.2	1.5	<0.001	0.04
Trunk–foot distance (% thigh length)	0.7	0.2	2.0	=0.153	0.01
Knee flexion angle (°)	1.0	0.9	1.0	=1	0.09
vGRF (N/kg)	1.1	0.9	1.3	=1	0.00
Early KAM (N m/kg)	2.0	0.1	65.0	= 1	0.00
Highest KAM (N m/kg)	0.3	0.0	2.9	= 1	0.00

*Note*: ORs are for a unit change in the listed variable. *r* ^2^ is the percentage variance explained by the fixed effect. The *p* values were adjusted (*3) to counter familywise comparisons. Trunk–foot distance is the mediolateral and anteroposterior distance between the foot and trunk segment centres of mass. Kinematics are extracted at initial contact. The vGRF and early KAM were extracted at their highest value during the first 100 ms after initial contact. The highest KAM was the maximum value over the entire stance phase.

Abbreviations: CI, confidence interval; KAM, knee abduction moment; OR, odds ratio; vGRF, vertical ground reaction force.

## DISCUSSION

The principal finding of this study shows evidence in support of the early peak waveform, identified with cluster analysis, as a risk factor for later sustaining ACL injury. Analysis of the 1776 trials of cutting manoeuvres performed by 84 athletes showed that the only other variable associated with a greater risk of ACL injury was the knee abduction angle at initial contact. Notably, discrete peak values of the knee abduction moment, whether extracted within 100 ms of initial contact or for the full duration of stance, were not associated with the risk of later sustaining ACL injury.

The early peak waveform was a strong risk factor for ACL injury. The fundamental challenge that informs this article is the analysis of biomechanics in a manner that is congruent with the ACL injury mechanism. The ACL injury occurs quickly after ground contact [17], but during this time frame, there is significant variability regarding whether or not the knee demonstrates a peak abduction moment [34]. This is an important point because, when conducting statistical tests, it is necessary to have all recorded trials represented in the analysis, regardless of whether the joint moment is an abduction or adduction moment. Other researchers have seemingly approached this fundamental challenge primarily by extracting the highest knee abduction moment value during a selected timeframe [38] or the entire stance phase [22]. However, these later stance peak values do not adequately represent the all‐important early stance phase [34]. Taken together, these discrepancies may explain why previous works have shown very conflicting results regarding the knee abduction moment as a risk factor for ACL injury [14, 18, 19, 22, 38].

Previous work on the early peak knee abduction waveforms identified via cluster analysis has demonstrated associations between the early peak waveform and kinematics related to ACL injury [33], and that the frequency distribution of early peaks between sexes resembles the frequency distribution of ACL injury [31]. Moreover, the early peak waveform frequency is potentially modified by subjecting athletes to preventive programmes [32]. With the addition of the current study, the first direct evidence is provided in support of the early peak waveform of the knee abduction moment being predictive of the ACL injury itself. The analysis is well‐powered at over 90% observed power, and cross‐validation showed that the results are not overly affected by a single outlier, with the lower bounds of the confidence interval still considered clinically relevant. Despite the low number of participants with injuries, this finding appears robust and significant. Thus, although care must be taken to interpret the results, there is an abundance of evidence to support a large‐scale prospective investigation to replicate these findings.

Traditional methods of extracting single data points and their values from the time series rather than waveform shapes based on multiple data points were also performed in the current study, but these peak values failed to show a clear association with the risk of ACL injury. A large recently published study used peak values extracted from the entire stance phase and found no association with ACL injury risk [22]. The authors did not state a reason for using the entire stance phase, but the results are consistent with another study that does look at the early stance phase values [38]. Several factors can affect the magnitude of the knee abduction moment and need consideration when interpreting research results or determining methods of data capture and processing. The first is measurement error either from inaccuracy in the placement of markers [23] or due to soft tissue artefacts [35, 36]. Notably, errors in marker placement have been shown to only have minor effects on the early peak waveform [33], and the low filtering frequency used in the present study would reduce soft tissue artefacts further. Second, the speed of the movement task and the sampling rate capturing the movement may play a role. Considering the narrow injury timeframe, a low sampling rate may miss relevant data. A task performed from a ready position, as was done in the present study, may produce a fairly low‐intensity task, whereas others have tried to emulate game‐like intensity [22, 38]. Similar to how every egg will crack if the fall is high enough, higher intensity movements may shift every athlete towards more knee abduction moment loading, whereas lower intensity movements may identify athletes with more extreme knee abduction loading tendencies.

The results regarding discrete peak values of the knee abduction moment within 100 ms must be viewed considering that the analysis was not powered to claim any effect because the upper bounds of the confidence interval were at a 65‐fold risk of ACL injury for each 1 N m/kg increase. The story changed somewhat when examining the peak values extracted over the entire stance phase as the upper limit of the confidence interval was dramatically lower or a threefold risk for each 1 N m/kg increase, which, considering that the difference between the lowest and highest values was only about 1.4 N m/kg, is not a very large effect. The values of the two extracted maximum values with the different methods were not interchangeable, as the mean difference in the timing of each variable was about 118 frames, or 295 ms. Although it is possible that higher movement speeds will result in the highest knee abduction moment values being observed in the early stance phase, the data of the present study generally found the highest values to be present in the late stance phase, which is not relevant for ACL injury risk.

The knee abduction angle was related to the risk of ACL injury, which is consistent with video analyses of ACL injuries in sports [8, 20]. Neither the knee flexion angle nor the vertical ground reaction force, representing stiff landings, were related to the risk of ACL injury in our cohort despite being considered an important component of ACL injury [5]. As the compression forces between the tibia and femur increase, the posterior slope of the tibial plateau causes the tibia to slide anteriorly relative to the femur, thus loading the ACL [3, 37]. As the knee is flexed, the orientation of the tibial slope should change and provide a load‐reducing effect on the ACL. These variables, obtained during a vertical drop jump, have also previously been shown to be related to the risk of ACL injury [19]. One explanation for our finding is the low‐speed approach and that the habitual movement pattern of the at‐risk athletes does not necessarily include a stiff landing, which may also primarily occur during in‐game situations. The same can be said regarding the trunk–foot distance, which is known to influence the knee abduction moment [33] and has been named as a potential risk factor [30], although some have questioned its importance [15]. Alternatively, the current study might not be powered to exclude these effects indicated by the large confidence intervals of the estimates, which include clinically meaningful values.

Based on the results of this study, future intervention studies may use the early knee abduction moment waveform as a marker for the efficacy of selected training strategies, with a focus on muscle strength and activation [28] or kinematics and movement patterns [33].

### Strengths and limitations

The movement task in the current study was a cutting manoeuvre from a ready position so the physical demands placed on the athlete during the direction change are rather low. Whether this is a strength or a limitation is a subject of debate. On the one hand, the athletes who demonstrate risky movement patterns under a low‐intensity movement are perhaps those who are most likely to have even more severe issues on the pitch. On the other hand, a low‐intensity movement is further from the actual sporting event and may therefore not reflect a sport‐specific movement pattern.

The signal processing used was quite extensive, including a low‐pass filter with a 6 Hz cut‐off. The choice of filter frequency was made due to the clustering algorithm working best when curve shapes are simplified through filtering. The magnitude of impact knee moments is reduced by this, but not lost as evidenced by early peak knee abduction moment signals. However, the true frequency of the early peak knee abduction moments may be higher.

The most important limitation is the sample size, which in the current study is low with only seven athletes having sustained ACL injuries. With a mixed logistic regression model, an a priori power calculation is not possible. We have addressed this limitation by providing the observed power of the models, but this is only an estimate and is based on the statistical model itself. Although the observed power supports the conclusions presented here and the findings are congruent with the expected theory of the injury mechanism, the risk of false discovery is likely fairly high.

Some of the uninjured athletes may have discontinued sports after the data collection, and therefore would not have injured their ACL. Because we did not follow up with every athlete, there may be a washout effect where some of those athletes who are truly at risk do not suffer the injury due to sports discontinuation. Our results may therefore be biased towards underestimating the strength of risk factors.

## CONCLUSION

This is the first study to find that the early peak knee abduction moment waveform was associated with the risk of ACL injury and may represent a novel and strong independent risk factor for ACL injury. If these findings can be replicated in a larger cohort, the early peak knee abduction moment may be used to screen athletes for risk and guide targeted preventive interventions. Although these findings are exploratory and hypothesis generating, we conclude that there is ample evidence to justify a large‐scale prospective study to confirm these findings.

## AUTHOR CONTRIBUTIONS

All authors contributed to the data collection and writing of the manuscript, and data processing and analyses were led by Haraldur Björn Sigurðsson.

## CONFLICT OF INTEREST STATEMENT

The authors declare no conflict of interest.

## ETHICS STATEMENT

This study was approved by the National Ethics Committee as noted in the manuscript (12‐040‐V8), with all participants (and guardians when appropriate) confirming informed consent before data collection.

## Supporting information

Supplementary information.

Supplementary information.

## Data Availability

The data used, as well as the R scripts to generate the results, will be made available by reasonable request to the corresponding author.
