# Supplementary material for: The early peak knee abduction moment waveform is a novel risk factor predicting anterior cruciate ligament injury in young athletes: A prospective study
Source: Knee Surg Sports Traumatol Arthrosc. 2024 Sep 12;33(5):1677–85. doi: 10.1002/ksa.12471 (PMC12022829; doi:10.1002/ksa.12471)
Supplement: Supplementary file 1 — Supplementary information. [file KSA-33-1677-s001.docx]

**Supplement Table:** Early peak waveform – Leave-one-out cross validation of model stability

95% CI

OR Lower Upper p-value

7.3 2.4 24.7 < 0.001

20.5 5.1 116.4 < 0.001

7.3 2.4 24.8 < 0.001

7.4 2.5 25.2 <0.001

5.0 1.5 18.4 = 0.007

8.0 2.1 38.8 = 0.002

3.8 1.1 13.9 = 0.030

7.3 2.4 24.8 < 0.001

Abbreviations: CI = confidence interval, OR = Odds ratio of ACL injury for trials with an early peak waveform compared to no early peak waveform.
