# Supplementary material for: The early peak knee abduction moment waveform is a novel risk factor predicting anterior cruciate ligament injury in young athletes: A prospective study
Source: Knee Surg Sports Traumatol Arthrosc. 2024 Sep 12;33(5):1677–85. doi: 10.1002/ksa.12471 (PMC12022829; doi:10.1002/ksa.12471)
Supplement: Supplementary file 2 — Supplementary information. [file KSA-33-1677-s002.docx]

STROBE Statement—checklist of items that should be included in reports of observational studies

|  | Item No. | Recommendation | Page  No. | Relevant text from manuscript |
| --- | --- | --- | --- | --- |
| **Title and abstract** | 1 | (*a*) Indicate the study’s design with a commonly used term in the title or the abstract | 1,2 | ‘Prospective’ stated in title and abstract |
|  |  | (*b*) Provide in the abstract an informative and balanced summary of what was done and what was found | 2 | Clearly stated in abstract. |
| Introduction | | | |  |
| Background/rationale | 2 | Explain the scientific background and rationale for the investigation being reported | 3 | Mechanism and timing of injury and relevance to data collection and processing is explained. |
| Objectives | 3 | State specific objectives, including any prespecified hypotheses | 4 | Two specific aims provided, see comment on hypotheses at top of Methods section |
| Methods | | | |  |
| Study design | 4 | Present key elements of study design early in the paper | 4 | First paragraph of methods states that this is a cohort study |
| Setting | 5 | Describe the setting, locations, and relevant dates, including periods of recruitment, exposure, follow-up, and data collection | 4-5 | Dates, location/setting, data collection and follow up information provided. |
| Participants | 6 | (*a*) *Cohort study*—Give the eligibility criteria, and the sources and methods of selection of participants. Describe methods of follow-up  *Case-control study*—Give the eligibility criteria, and the sources and methods of case ascertainment and control selection. Give the rationale for the choice of cases and controls  *Cross-sectional study*—Give the eligibility criteria, and the sources and methods of selection of participants | 4-6 | Eligibility (having follow up data, providing consent and having continued sports) information provided, as was further data collection, and choice of case (injured knees) and controls (uninjured knees). |
|  |  | (*b*) *Cohort study*—For matched studies, give matching criteria and number of exposed and unexposed  *Case-control study*—For matched studies, give matching criteria and the number of controls per case | 6-7 | Number of injuries and injured knees provided |
| Variables | 7 | Clearly define all outcomes, exposures, predictors, potential confounders, and effect modifiers. Give diagnostic criteria, if applicable | 5-6 | All relevant variables defined, incl.random vs. fixed effects, as are confounders (and in discussion) |
| Data sources/ measurement | 8* | For each variable of interest, give sources of data and details of methods of assessment (measurement). Describe comparability of assessment methods if there is more than one group | 4-5 | Data collection and processing described in detail. |
| Bias | 9 | Describe any efforts to address potential sources of bias | 4 | N/A |
| Study size | 10 | Explain how the study size was arrived at | 4 | Previous participants contacted for participation |

| Quantitative variables | 11 | Explain how quantitative variables were handled in the analyses. If applicable, describe which groupings were chosen and why | 4-5 | Methods on data collection and processing clearly described |
| --- | --- | --- | --- | --- |
| Statistical methods | 12 | (*a*) Describe all statistical methods, including those used to control for confounding | 6-7 | All stats for primary and secondary analyses explained |
|  |  | (*b*) Describe any methods used to examine subgroups and interactions | N/A |  |
|  |  | (*c*) Explain how missing data were addressed | N/A |  |
|  |  | (*d*) *Cohort study*—If applicable, explain how loss to follow-up was addressed  *Case-control study*—If applicable, explain how matching of cases and controls was addressed  *Cross-sectional study*—If applicable, describe analytical methods taking account of sampling strategy | 6-7 | See previous comments on inj/uninj |
|  |  | (*e*) Describe any sensitivity analyses | 6 | Cross-validation |
| Results | | | | |
| Participants | 13* | (a) Report numbers of individuals at each stage of study—eg numbers potentially eligible, examined for eligibility, confirmed eligible, included in the study, completing follow-up, and analysed | 4  6 | Demographic data provided  Injury data provided |
|  |  | (b) Give reasons for non-participation at each stage | 4 | Based on consent at each stage |
|  |  | (c) Consider use of a flow diagram |  | Preferred use of main text |
| Descriptive data | 14* | (a) Give characteristics of study participants (eg demographic, clinical, social) and information on exposures and potential confounders | 4-5 | See methods - participants |
|  |  | (b) Indicate number of participants with missing data for each variable of interest | N/A |  |
|  |  | (c) *Cohort study*—Summarise follow-up time (eg, average and total amount) |  |  |
| Outcome data | 15* | *Cohort study*—Report numbers of outcome events or summary measures over time |  |  |
|  |  | *Case-control study—*Report numbers in each exposure category, or summary measures of exposure | 7 | Top of results |
|  |  | *Cross-sectional study—*Report numbers of outcome events or summary measures |  |  |
| Main results | 16 | (*a*) Give unadjusted estimates and, if applicable, confounder-adjusted estimates and their precision (eg, 95% confidence interval). Make clear which confounders were adjusted for and why they were included | 7 | OR with 95% CI provided for overall risk of ACL injury, and for the cross validation. Mixed logistic regression for secondary analysis also provided 95% CIs and adjusted p-values as noted in methods |
|  |  | (*b*) Report category boundaries when continuous variables were categorized |  |  |
|  |  | (*c*) If relevant, consider translating estimates of relative risk into absolute risk for a meaningful time period |  |  |

| Other analyses | 17 | Report other analyses done—eg analyses of subgroups and interactions, and sensitivity analyses | 8 | Analysis of discrete values of specific variables reported (secondary aim). |
| --- | --- | --- | --- | --- |
| Discussion | | | | |
| Key results | 18 | Summarise key results with reference to study objectives | 9 | First paragraph of Discussion summarizes principal findings. |
| Limitations | 19 | Discuss limitations of the study, taking into account sources of potential bias or imprecision. Discuss both direction and magnitude of any potential bias | 11 | Limitations, strengths, risk of false discovery, and underestimation of risk are discussed. |
| Interpretation | 20 | Give a cautious overall interpretation of results considering objectives, limitations, multiplicity of analyses, results from similar studies, and other relevant evidence | 9-11 | Results are interpreted and discussed within the context of few injuries, methods used, other publications |
| Generalisability | 21 | Discuss the generalisability (external validity) of the study results | 11 | Discussed in Limitation section |
| Other information | |  | | |
| Funding | 22 | Give the source of funding and the role of the funders for the present study and, if applicable, for the original study on which the present article is based | Title page |  |

*Give information separately for cases and controls in case-control studies and, if applicable, for exposed and unexposed groups in cohort and cross-sectional studies.

**Note:** An Explanation and Elaboration article discusses each checklist item and gives methodological background and published examples of transparent reporting. The STROBE checklist is best used in conjunction with this article (freely available on the Web sites of PLoS Medicine at http://www.plosmedicine.org/, Annals of Internal Medicine at http://www.annals.org/, and Epidemiology at http://www.epidem.com/). Information on the STROBE Initiative is available at www.strobe-statement.org.
